# Neutral theory and the species abundance distribution: recent developments and prospects for unifying niche and neutral perspectives

**DOI:** 10.1002/ece3.1092

**Published:** 2014-05-02

**Authors:** Thomas J Matthews, Robert J Whittaker

**Affiliations:** 1 Conservation Biogeography and Macroecology Programme, School of Geography and the Environment, University of OxfordSouth Parks Road, Oxford, OX1 3QY, UK; 2 Azorean Biodiversity Group (ABG CITA-A) and Portuguese Platform for Enhancing Ecological Research and Sustainability (PEERS), Departamento de Ciências Agrárias, University of the AzoresRua Capitão João d′Ávila, Pico da Urze, 9700-042, Angra do Heroísmo, Portugal; 3 Center for Macroecology, Evolution and Climate, Department of Biology, University of CopenhagenUniversitetsparken 15, DK-2100, Copenhagen Ø, Denmark

**Keywords:** Emergent neutrality, neutral theory, niche theory, species abundance distribution, stochastic niche theory

## Abstract

Published in 2001, The Unified Neutral Theory of Biodiversity and Biogeography (UNTB) emphasizes the importance of stochastic processes in ecological community structure, and has challenged the traditional niche-based view of ecology. While neutral models have since been applied to a broad range of ecological and macroecological phenomena, the majority of research relating to neutral theory has focused exclusively on the species abundance distribution (SAD). Here, we synthesize the large body of work on neutral theory in the context of the species abundance distribution, with a particular focus on integrating ideas from neutral theory with traditional niche theory. First, we summarize the basic tenets of neutral theory; both in general and in the context of SADs. Second, we explore the issues associated with neutral theory and the SAD, such as complications with fitting and model comparison, the underlying assumptions of neutral models, and the difficultly of linking pattern to process. Third, we highlight the advances in understanding of SADs that have resulted from neutral theory and models. Finally, we focus consideration on recent developments aimed at unifying neutral- and niche-based approaches to ecology, with a particular emphasis on what this means for SAD theory, embracing, for instance, ideas of emergent neutrality and stochastic niche theory. We put forward the argument that the prospect of the unification of niche and neutral perspectives represents one of the most promising future avenues of neutral theory research.

## A Brief History of Neutral Ecology

The dominant view in ecology in the first half of the twentieth century was that of stability and of ecological communities structured through mechanisms such as competitive interactions, density dependence, local adaptations, and niche differentiation (Begon et al. 1990). This paradigm was challenged through various theories published in the mid-twentieth century, such as MacArthur and Wilson's (1967; see Whittaker and Fernández-Palacios 2007) equilibrium theory of island biogeography (ETIB), with researchers looking at broader-scale structuring mechanisms. More recently Hubbell (2001) generated substantial debate when he challenged the traditional niche-based view of community structure by claiming that numerous macroecological patterns, including species abundance distributions (SADs; see Table 1 for definitions of key terms and acronyms used in this article) and species–area relationships (SARs), could be explained through his Unified Neutral Theory of Biodiversity and Biogeography (UNTB). Following Caswell (1976), Hubbell (2001, 2006) put forward the argument that species differences are not necessarily evidence of important roles for niche differentiation, criticizing the “uncritical acceptance” of such evidence in the ecological literature. Thus, neutral theory rejects two concepts that have formed the basis of traditional ecological research and niche theory (Chesson 1991), namely that species are ecologically and functionally different, and that environmental context is important (McGill 2006; Rosindell and Cornell 2007). In this regard, neutral theory is frequently misinterpreted. It does not imply that species are undifferentiated in their traits. Rather it asks the questions: do these differences matter in terms of ecological community structure, and are they functionally significant?

**Table 1:** Neutral theory and species abundance distribution terminology

Term	Definition
Emergent neutrality (EN)	A model in which neutrality is the outcome of community evolution. According to the model competing species self-organize into groups of species with similar traits
Fundamental biodiversity number	A dimensionless parameter in neutral models which describes various characteristics of the metacommunity. The parameter (*θ*) is given by 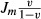 , where J_m_ is the number of individuals in the metacommunity, and *v* is the probability that a deceased individual is replaced by a new species through speciation (per capita speciation rate)
Fundamental immigration number	A parameter (I) given by 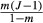 , where J is the number of individuals in the local community and *m* is the probability that a deceased individual in the local community is replaced by an immigrant from the metacommunity. It is often used in neutral models as a measure of dispersal limitation as the parameter *m* can be difficult to interpret. “I” is also independent of sample size and can be seen as measure of community isolation
Likelihood surface	The value of the likelihood, usually displayed in graphical form, as a function of a number of parameters (generally two or three). Multiple local maxima refer to the situation in which the likelihood surface has more than one peak
Local community	Generally relates to the local community in Hubbell's spatially implicit neutral model, in which it is a set of individuals that live in the same smaller sample/island distinct from the larger metacommunity/mainland. A dead individual is immediately replaced either by an offspring of another individual (of any species) in the local community, or by an immigrant from the metacommunity (determined by *m*; the probability that a deceased individual in the local community is replaced by an immigrant from the metacommunity). As long as *m* is greater than 0 the local community receives a certain amount of immigrants from the metacommunity. The number of individuals in the local community is predicted to be too small for speciation to occur
Lognormal distribution	A probability distribution of a random variable whose logarithm follows a Gaussian distribution. In relation to species abundance distributions, the lognormal distribution characterizes a sample with relatively few very abundant or very rare species
Logseries distribution	A probability distribution which results from the Poisson sampling of a gamma distribution after a certain relevant limit is taken, and conditional presence is considered, that is, it gives the conditional probability of attaining a certain abundance level given that the species is present. In relation to species abundance distributions, the logseries distribution characterizes a sample in which the most common abundance category is a single individual
Metacommunity	In the context of neutral theory, the metacommunity generally relates to Hubbell's spatially implicit neutral model, in which it is the source pool of individuals. A dead individual is immediately replaced either by an offspring of another organism in the metacommunity, or by an individual from a new species (speciation). Offspring of individuals may disperse to the local community (above)
Multimodal species abundance distribution	A species abundance distribution with multiple modal abundance values or octaves. The majority of published species abundance distribution models are unimodal, but it has become increasingly apparent that many empirical abundance distributions exhibit multiple modes
Spatially explicit neutral model (SENM)	A neutral model that incorporates an explicit spatial structure, which enables the model to predict the exact location of each individual in space
Spatially implicit neutral model (SINM)	A neutral model that incorporates a restricted consideration of spatial structure. Hubbell's (2001) classic neutral model is spatially implicit as it only focuses on two scales of community organization, that is, the metacommunity and the local community
Speciation mode (within neutral models)	The manner in which speciation is modelled in neutral models. In Hubbell's classic SINM, speciation occurs via the point mutation mode whereby speciation is an instantaneous process. Neutral models incorporating alternative speciation modes have since been developed; for instance, whereby speciation is a gradual, drawn out process (protracted speciation)
Species abundance distribution (SAD)	The typical univariate SAD gives the expected frequency of species at each abundance level, either in terms of relative frequencies or simply by the average number of species at each abundance level. The multivariate SAD gives the whole multidimensional distribution: the abundance of all species observed within a sample of an ecological community
Species–area relationship (SAR)	The relationship between the area of a sample or island and of the number of species in that area
Stochastic niche theory	A theory of community structure which combines niche apportionment with stochastic processes
Zero-sum assumption	An assumption of many neutral models, including Hubbell's (2001) SINM, stipulating that when an individual dies it is immediately replaced by another individual, that is, resources are fully saturated at all times
Zero-sum multinomial distribution (ZSM)	The species abundance distribution predicted for the local community in Hubbell's (2001) SINM (above)

Hubbell (2001) evaluated the UNTB by means of an extensive analysis of a dataset from Barro Colorado Island (herein “BCI”). This dataset is a count of all tree and shrub species greater than 1 cm in stem diameter from a 50-ha plot. He found that by focusing on all species it was possible to observe significant differences between species, implying niche differentiation, but that 75% of the species occupied a similar shade-tolerant niche. Hubbell (2001, 2006) argued that niche-based theories did not provide explanations for the multitude of such species; it being more likely that such a pattern arose through the abundance of shady habitat compared with open habitats over the evolutionary history of the species. Thus, these species have simply evolved life histories adapted to shady habitats regardless of other species life histories. This has led to the coexistence of ecologically equivalent species (Hubbell 2006), a pattern seemingly explained through the UNTB. Furthermore, as individuals in species-rich communities frequently have numerous different nearest neighbors relative to conspecific individuals (an argument constructed by reference to plants), any local adaptation is unlikely to be along the same direction between individuals. This, it was argued, led not to niche divergence and specialist life histories but to niche convergence towards generalist strategies and, therefore, ecological equivalence (Hubbell and Foster 1986; Hubbell 2006).

Neutrality was not a new concept, having been applied in the field of population genetics during the 1960s (e.g., Kimura and Crow 1964; Kimura 1968; King and Jukes 1969; Watterson 1974a; for a review see Leigh 2007). In this context, neutrality refers to a situation where there is equal likelihood that a gene enters the next generation irrespective of its allelic type. The theory was subsequently applied to ecology (e.g., Watterson 1974b; Caswell 1976). However, whereas these previous applications of neutral theory had considered the importance of random genetic drift, Hubbell also postulated that random dispersal was a primary controlling factor of community structure, that is, the UNTB is a dispersal-assembled theory (Hubbell 2001). The UNTB asserts that species abundances result from a combination of dispersal, speciation, and stochastic variation in birth and death rates (ecological drift). Thus, the abundance of species changes by chance and not because of differences in competitive ability (Hubbell 2001; Etienne and Olff 2004). Extinction is also an important process but neutral models assume that extinction rate can be predicted if values of the other processes are known (Borda-de-Água et al. 2007). In Hubbell's (2001) original work, dispersal was modeled in various ways. For instance, in Chapter 5, dispersal was based on the “voter model,” in which species can only disperse to sites immediately adjacent to those they occupy at each given time step. Such a model is unrealistic and subsequent work (Borda-de-Água et al. 2007; see also Rosindell and Cornell 2013) has advanced this approach to incorporate dispersal kernels (“Lévy flights”) that allow for long-distance dispersal. Neutral theory also assumes that as species are functionally equivalent, the rates of these processes are the same for each species on a per capita basis and that variation in abundance between species is a result of “accidental” dispersal and ecological drift (Hubbell 2001; Holt 2006; Borda-de-Água et al. 2007). Hubbell (2001) originally contended that the theory should be applicable to multiple taxa; albeit only when considering trophically similar species.

Neutrality can be condensed into two forms: hard and weak. Weak neutrality describes the situation in which null models moderately explain community structure, even if fine scale examination reveals that species do differ in their niche characteristics. Hard neutrality relates to a situation whereby species are completely functionally equivalent and species identity does not matter in explaining macroecological patterns, such as SADs (Bell 2001; Holt 2006). Furthermore, around 10 different variants of neutral models have been discussed in the ecological literature, each with slightly different predictions for different factors. Hubbell's (2001) neutral model is a spatially implicit neutral model (SINM) that focuses on two scales of community organization: the metacommunity (source pool of individuals) and the local community. This model is also referred to as the mainland–island model, where the metacommunity represents the mainland source pool of individuals with the potential to disperse to the island community. Recent work by Rosindell and colleagues (e.g., Rosindell et al. 2008; Rosindell and Cornell 2013) has developed spatially explicit neutral models (SENM) in which the two-tier community hierarchy (meta- and local community) is replaced with a multiscale structure. While authors often use the term “neutral theory” to describe particular models (e.g., Hubbell's SINM), we agree with Rosindell et al. (2012) that the term should refer to the larger collection of neutral models.

Although the present review is focused largely on SADs, the application of neutral theory is not restricted to SADs, and numerous macroecological patterns have been explored using neutral models, such as species–area relationships (SARs), abundance–occupancy relationships, species turnover, and the distance-decay relationship (Bell 2001; Chave and Leigh 2002). This conceptual breadth is one of the advantages of the theory.

A number of other reviews of neutral theory exist (e.g., Alonso et al. 2006; Leigh 2007; Rosindell et al. 2011). However, these approach neutral theory in a more general way than our review. Here, our purpose is to provide an in-depth analysis of the species abundance distribution in the context of neutral theory. Furthermore, past reviews have focused solely on neutral theory proper (e.g., Rosindell et al. 2011), rather than on the idea of unifying neutral and niche perspectives, and as such do not cover recent advancements such as stochastic niche theory and emergent neutrality. A considerable amount of research has been undertaken on integrating the aspects of niche and neutral theories in the last 10 years, and we feel a review of this material may provide a useful resource which (1) condenses the considerable amount of work that has already been published and (2) provides a catalyst and guide for future work in this direction.

## Neutral Theory and the Species Abundance Distribution

A multivariate species abundance distribution (herein “SAD”) describes the abundances of all species sampled within a given community (Table 1; Alonso et al. 2008; Ulrich et al. 2010). To date around 30 different SAD models have been published (McGill et al. 2007). The two most commonly used are the logseries (Fisher et al. 1943) and the lognormal (Preston 1948). The logseries distribution results from the Poisson sampling of a gamma distribution: the modal abundance value in a logseries distribution is one (Fisher et al. 1943). The lognormal distribution represents a situation in which the logarithms of the different species’ abundances follow a Gaussian distribution, and as such it characterizes a community with relatively few very abundant or very rare species.

**Figure 1:**
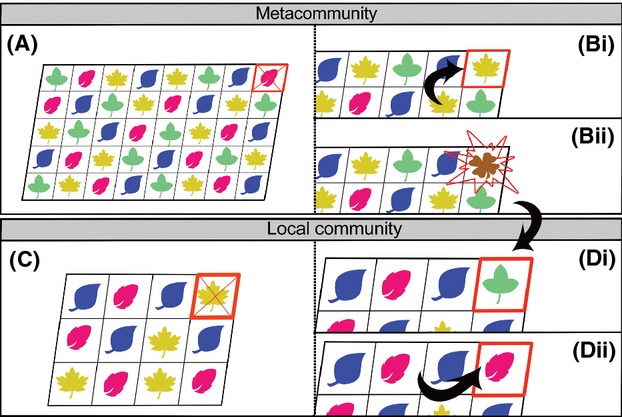
An illustration of Hubbell's (2001) classic two-tier spatially implicit neutral model. The different colored leaves represent different species of trees. The top row (A & B) represents the metacommunity (a large continuous forest which is the source pool of individuals) and the bottom row (C & D) represents the local community (a smaller distinct patch of forest). In the metacommunity (A), at time step *t* a random individual (highlighted by a red square and cross) dies and is instantly replaced at time step *t* + 1 (the zero-sum assumption) by either the offspring of another individual in the community (Bi; with probability 1−*v*) or through the instantaneous emergence (point mutation) of a new species, that is, speciation occurs (Bii; with probability *v*). A similar process occurs in the local community, but here immigration replaces speciation. At time step *t* a randomly chosen individual dies (C; highlighted by a red square and cross) and is instantly replaced by either an immigrant from the metacommunity (Di; with probability *m*), or via the offspring of any species in the local community (Dii; with probability 1−*m*), at t + 1. The local community is generally assumed to be panmictic, that is, dispersal limitation is ignored and any individual has the same probability of producing the offspring that replaces the deceased individual.

Hubbell's (2001) general argument in relation to SADs was that the lognormal gives an underestimate of the number of rare species in species-rich communities; that is, it did not deal with the log-left skew observed in many natural systems (Gray et al. 2006). In Hubbell's SINM, the relative abundance of species within – and the species diversity of – a community can be explained through neutral drift of individual species’ abundances. The model contends that the number of individuals in a metacommunity (J_m_) is constant, that is, all available resources in the community are saturated. This is the zero-sum assumption: if an individual dies and a portion of the resource becomes available, it will be immediately taken up by a new individual, and the community size remains constant (Hubbell 2001). Certain studies have relaxed the zero-sum assumption (e.g., Volkov et al. 2003; Etienne et al. 2007a; see discussion in Alonso et al. 2006) but it has been found that this does not change the form of the SAD (Etienne et al. 2007a). The deceased individual is replaced either via a new species through speciation (with probability *v*) or via the offspring of a randomly selected individual from a species already present in the community (probability 1−*v*; see Fig. 1 for an illustration of this process). The assumption of neutrality implies that these probabilities (i.e., that an individual undergoes speciation, or reproduces) are identical for all species in the community on a per capita basis. The SAD can be determined using what Hubbell termed the “fundamental biodiversity number” (*θ*), whereby 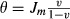, or in the case of nonoverlapping generations *θ* = 2J_m_*v*. This dimensionless biodiversity parameter is independent of sample size, specifying the number of new species that appear in the community on average per generation, the number of predicted species present in the metacommunity at a steady state between extinction and speciation, and the predicted abundance of each species at metacommunity scale. The parameter is also proportional to both the size of the metacommunity and the average per capita metacommunity speciation rate (Hubbell 2001; He and Hu 2005; Hubbell et al. 2008). It has also transpired that *θ* is equivalent to Fisher's *α* and the expected SAD for the metacommunity is Fisher et al.'s (1943) logseries (described above; see also Watterson 1974b; Hubbell et al. 2008). The parameter *θ* has also been found to have an analytical relationship with Simpson's diversity index (He and Hu 2005).

**Figure 2:**
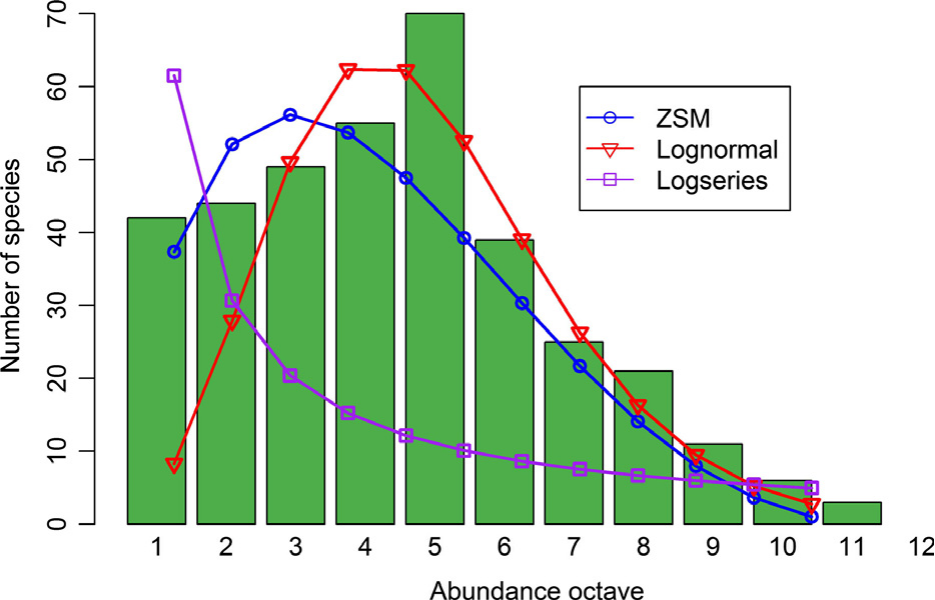
Exemplar fits of three species abundance distribution models: the zero-sum multinomial distribution of Hubbell's (2001) spatially implicit neutral model, the Poisson lognormal distribution and the logseries distribution (Fisher et al. 1943). The models are fitted to simulated data (green bars; 365 species and 22945 individuals). The three models are fitted using maximum likelihood methods. The simulated data are binned into octaves following method 3 in Gray et al. (2006): the first octave contains the number of species represented one individual, the second octave contains the number of species with 2–3 individuals, the third octave represents 4–7 individuals, and so on. The asymmetry of the ZSM enables it to provide a better fit than the other distributions to the left hand tail of the empirical distribution.

In the SINM, the local community is embedded within the wider metacommunity, with the former often provided with immigrants from the latter. At the local community scale (population size J), there is no speciation and, at the death of an individual, it is replaced either by a randomly selected immigrant from the metacommunity (probability of *m*) or via the offspring of a random individual of a species already present within the local community (probability of 1−*m*; see Fig. 1). If there is no immigration into the local community from the metacommunity (i.e., *m =* 0), then the SINM predicts that eventually only one species will remain (termed “monodominance”), as all others are lost through a random walk to extinction. The “fundamental dispersal number (I),” calculated as 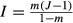 (for nonoverlapping generations *I* = 2J*m*), is often used because interpreting *m* as a measure of dispersal limitation is problematic. Hubbell's (2001) SINM predicts that the SAD at the local community level will follow a zero-sum multinomial distribution (ZSM; also termed the dispersal limited multinomial by later studies, for example, Etienne and Alonso 2005). The ZSM is parameterized by *θ*, *m*, and J (Hubbell 2001). The parameter *m* is central in the shaping of the SAD of the local community and as *m* approaches zero (low migration communities) the local community supports fewer rarer species as it progresses toward monodominance. For a given value of *θ*, a high migration system (*m* > 0.1) will possess higher local diversity than a low migration system (*m* < 0.1); the latter always having higher turnover (Latimer et al. 2005). At *m* = 0 evidently only one species will be present, but at intermediate values of *m* the local community SAD will have a lognormal type shape. Thus, shifts in the value of *m* offer an explanation for the log-left skew of the standard lognormal form that has been observed in many ecological studies (i.e., there are often more rare species than predicted by the lognormal; see Fig. 2 for an example of the fitted ZSM), and indeed the prevalence of logseries distributions (Bell 2001; Magurran 2005). The ability of the ZSM to unite the shapes of the lognormal and logseries distributions results in the good fit to empirical data. Nonetheless, it has been noted that *m* and *θ* can also be seen as purely “geometric descriptors” of the shape of the distribution without any assumption of the validity of the neutral model that underpins them (McGill et al. 2006; McGill 2011). There is also an issue in using maximum likelihood methods to estimate the parameter values, as while this approach may produce parameter values that result in a good statistical fit, the estimated values may be ecologically unrealistic (Gotelli and McGill 2006; McGill et al. 2006).

The shape of the SAD has also been explored using SENMs. For instance, Rosindell and Cornell (2013) investigated the SADs predicted by an SENM at multiple spatial scales. SADs from the SENM were found to be parameterized by a single parameter equivalent to the ratio A/R, whereby A is the number of individuals in the sample and R is proportional to a species’ spatial range (Rosindell and Cornell 2013). As with the SINM, the SENM predicts a logseries SAD at the largest scales. However, the predicted shape of the SAD at scales smaller than the metacommunity differs from that of the SINM.

The mode of speciation (Table 2) on which a model is based can also affect the shape of the SAD under neutral dynamics. Hubbell's (2001) monograph was largely based on the idea of point mutation. Except perhaps for speciation by polyploidy, point mutation is unrealistic as it implies a proportional relationship between abundance and rate of speciation (Etienne et al. 2007b). However, in a comparison of neutral models where speciation rate per species was proportional to abundance and where speciation was independent of abundance using 20 tree community datasets, Etienne et al. (2007b) found that datasets are better fitted by the point mutation models. This issue may have since been resolved as it has been found that a neutral model incorporating protracted speciation (Table 2) is more ecologically realistic and still retains a good fit to SAD data.

**Table 2:** The different speciation modes that have been incorporated into neutral models

Speciation Mode	Synopsis	References
Point mutation	Speciation whereby each individual in the metacommunity has an equal probability of producing an offspring of a new species. Produces many rare species with lifetimes unrealistically short	Hubbell (2001)
Random fission	Speciation occurs through a population randomly dividing into two distinct species. Produces species with lifetimes unrealistically long	Hubbell and Lake (2003), Hubbell (2005), Etienne and Haegeman (2011)
Peripheral isolate	Divergence follows the isolation of populations. Newly arisen species have abundances drawn from a normal distribution	Hubbell and Lake (2003), Hubbell (2005)
Generalized speciation	A generalized neutral community model incorporating numerous modes	Haegeman and Etienne (2009)
Protracted speciation	Speciation is a gradual, drawn out process. Results in a new predicted metacommunity SAD (termed the “difference logseries”)	Rosindell et al. (2010)

## Criticisms of Neutral Models

Hubbell's (2001) publication resulted in a heated debate (e.g., McGill 2003; Ricklefs 2003, 2006; Leigh 2007; Clark 2012). This is perhaps unsurprising, given that the basic assumptions of the theory ostensibly challenge many of the foundational concepts within ecology. The criticisms can be condensed into four main themes: (1) underlying assumptions; (2) fitting the ZSM and interpreting the parameters; (3) changes through time; and (4) attributing process to patterns. Each of these thematic criticisms will be discussed in turn.

A major issue is that species adaptations are known to be important over varying spatial scales (e.g., Condit et al. 1996; Engen et al. 2002), and deterministic processes of species interactions are known, at least partially, to determine species distributions. Even proponents of the neutral approach (e.g., Bell 2001; Hubbell 2001) agree that the theory fails at spatial scales where adaptations are important, that is, fine spatial scales (see Jabot and Chave 2011). The question then is not whether this assumption of neutral theory is true in all instances, as it evidently is not. Rather, the real question is given that the assumption is false, should neutral models and the SADs they predict be discarded? While, Chave (2004) suggests not, Ricklefs (2006) argues there is no escaping the falsified predictions involving ecological drift and changes through time (discussed below).

A specific issue relating to the SAD in the years immediately following Hubbell's (2001) monograph was the challenge of comparability of the fit of the ZSM with traditional SADs due to the complexity of the former's calculation. However, subsequent advances in regard to the ZSM have developed a sampling theory and derived its analytical form and likelihood function (e.g., Vallade and Houchmandzadeh 2003; Volkov et al. 2003; Alonso and McKane 2004; McKane et al. 2004; Etienne 2005; Etienne and Alonso 2007). The sampling formula of Etienne (2005; Table 3) was a particularly significant development and represents the most robust method of fitting the predicted SAD of the SINM to observed data and of generating the model likelihood. A summary of developments in fitting the ZSM, including several of the tests of the ZSM involving the BCI data, is presented in Table 3.

**Table 3:** A summary of the developments in the history of the zero-sum multinomial distribution (ZSM) of the spatially implicit neutral model (SINM), and the SAD of the spatially explicit neutral model (SENM), and the attempts to fit both to the Barro Colorado Island 50 ha tree dataset

Study Authors	Best Model/Main Finding	Subsequent Criticisms
Hubbell (2001)	ZSM	Goodness of fit only determined by graphical observation.
McGill (2003)	ZSM does not fit the data better than the lognormal	Used simulations to fit the ZSM
Volkov et al. (2003)	Derived an analytical solution for the ZSM and found the ZSM provided the best fit	Analytical equations did not represent the full solution as they applied solely to the mean number of species in a given class (Etienne and Olff 2004)
Vallade and Houchmandzadeh (2003)	Published a full analytical solution for the ZSM	Equations were later determined to be flawed (i.e., they applied the mean number of species in a given class) and were corrected by Etienne and Alonso (2005)
Alonso and McKane (2004)	Developed a different analytic solution	Rigorous fitting of the ZSM required likelihood methods
Etienne and Olff (2004)	Found slightly better support for the lognormal using a Bayesian approach	
Etienne (2005)	Published the correct analytical solution and sampling formula. Two forms of the likelihood equations exist: (a) Ewens’ (1972) sampling formula of neutral alleles is used in the case of no dispersal limitation and (b) Etienne's (2005) formula in cases of dispersal limitation	
Etienne and Alonso (2005)	Unified two different approaches to arrive at the full analytical solution: the genealogical approach (Etienne 2005) and master equation-based approach (e.g., Alonso and McKane 2004)	
McGill et al. (2006)	Compared nine goodness-of-fit measures with the BCI data and found that for eight out of the nine measures the lognormal outperformed the ZSM	
Jabot and Chave (2011)	Built on Etienne's (2005) maximum likelihood framework to develop a more robust test of neutrality incorporating the SAD of Hubbell's SINM and Shannon's index. The SAD of the BCI 50-ha plot did not significantly differ from neutrality; however, the SADs of smaller scale subplots from within the BCI plot were significantly non-neutral	
Rosindell and Cornell (2013)	(a) The gamma and negative binomial distributions provided a better fit than the ZSM (b) The SENM predicts SADs which are more realistic than those from the SINM	

McGill et al. (2006) outline two further issues with fitting the ZSM. First, as the ZSM has three parameters independent of each other, it is particularly flexible in terms of fitting, and a good fit to a particular dataset is not surprising. Second, and despite the biological meaning of the parameters being one of the original perceived benefits of neutral theory (Hubbell 2001), the parameters can be exceedingly difficult to calculate. While calculating the size of the local population (J) is an achievable aim, deducing the speciation rate (*v*) is likely a near impossible task (but see Ricklefs 2006), and calculation of the metapopulation size (J_m_) is contingent on being able to effectively delineate the metapopulation boundaries (Ricklefs 2003; McGill et al. 2006). However, it is important to remember that the ZSM is the SAD predicted by a particular neutral model, Hubbell's (2001) SINM. The recent finding of Rosindell and Cornell (2013) that the SADs of the SENM are more realistic than the ZSM may overcome many of these issues and warrants further study. For instance, SENMs are advantageous in regard to parameter estimation as they do not require calculation of the metapopulation size; but they do still require estimation of *v*.

Methodological problems were also found to impact the predictions of studies incorporating neutral dynamics (see Connolly and Dornelas 2011; Matthews and Whittaker 2014 in press for a discussion of methodological issues in SAD research more generally). For instance, Latimer et al. (2005) used an approximation of the likelihood function of Hubbell's neutral model in combination with Bayesian methods to answer questions related to immigration and speciation in the context of SADs in the Cape Floristic Region (CPF) biodiversity hotspot, South Africa. It was concluded that the neutral model can corroborate theories of high speciation rates and low migration within the region (Latimer et al. 2005). However, in a reply to this article, Etienne et al. (2006) argued that the exact likelihood function should be used as it enables more effective searching of the parameter space. For one of the three CPF datasets analyzed by Latimer et al. (2005), it was found that there were in fact two similar maxima in the likelihood and that using the exact likelihood resulted in different parameter values to those of Latimer et al. (Etienne et al. 2006). These multiple peaks in the likelihood relate to different ecological situations (i.e., different parameter values) and it is important to determine the actual maximum likelihood for a given study system (e.g., Connolly et al. 2009, Matthews & Whittaker 2014 in press).

A problem with neutral theory not confined to SADs is the predictions the theory makes relating to changes over time, particularly population changes through time and the life span of species (Leigh 2007). For instance, Ricklefs (2006) estimates that a European passerine species population of 14.43 × 10^6^ individuals with a generation time of 3 years would take more than 86 × 10^6^ years to become extinct under a neutral model of random drift. This is clearly too long based on the existing best knowledge of species life span estimates (Rosenzweig 1995; Ricklefs 2003, 2006). Thus factors other than random drift must be operating, such as changes in climate. That being said, most of this criticism has been directed to neutral models incorporating unrealistic modes of speciation. Incorporating protracted speciation into a neutral model has been found to greatly improve estimates of speciation rates and species lifetimes (Rosindell et al. 2010).

Finally, and as for many other ecological theories, the presence of a particular pattern does not provide proof of a particular process (McGill et al. 2007). Neutral patterns can arise from non-neutral mechanisms (Purves and Pacala 2005; Alonso et al. 2006). For example, complex ecological interactions in conjunction with variable natural conditions may prevent actual competitive differences between species being expressed, leading to nonequivalent species behaving neutrally (see Alonso et al. 2006). It has also been argued that diversity patterns such as the SAD are not diagnostic ecological tools (e.g., Leigh 2007; Clark 2012). The SAD, in addition to other diversity patterns such as the SAR, is an aggregated (i.e., macroecological) pattern in which the property of interest is characterized by integrating over a group of species. This aggregation has been criticized as resulting in a loss of information, which subsequently reduces the power of SADs to distinguish between communities that are influenced primarily by stochastic processes and those which are influenced by mainly deterministic (niche) processes (Chisholm and Pacala 2010; Clark 2012; see also discussion in Pueyo et al. 2007). For instance, it has been suggested that neutral patterns can emerge as a result of averaging over a group of species that are ecologically non-neutral and which individually may differ considerably from the average behaviour (Pueyo et al. 2007; Bowler and Kelly 2012). Thus, it is possible that the success of neutral theory in tests focused on aggregated patterns such as SADs and SARs may be explained in part by this averaging process (see Bowler and Kelly 2012); and thus the goodness of fit of SADs predicted by neutral models cannot be used as incontrovertible evidence for the absence of niche structure (Chisholm and Pacala 2010). That being said, SADs have been shown to provide useful information regarding community structure (e.g., Ugland and Gray 1982; Jabot and Chave 2011; Chust et al. 2013; Matthews et al. 2014), and as presence–absence and abundance data are often all that is available for many ecological datasets, we would argue that focusing on the SAD may still prove enlightening.

## The Other Side of the Coin: A Focus on Unifying Niche and Neutral Perspectives

Despite attracting much criticism, it may be argued that neutral theory is beneficial in that it allows for better testing and development of predictions surrounding community structure. It provides a dynamic sampling theory of community assembly founded on key processes, namely dispersal, speciation, birth, and death. Several studies have used neutral models to gain insights into the mechanisms driving diversity patterns. For instance, a neutral metacommunity model revealed the fundamental role of dispersal in generating spatial diversity patterns in the Mississippi–Missouri River System, and indicated that it was not necessary to incorporate species differences into the model to predict large-scale diversity patterns in this system (Muneepeerakul et al. 2008). For marine phytoplankton communities, plotting the *m* parameter of the SINM neutral model against latitude revealed that immigration probability is typically lower in tropical, relative to temperate communities (Chust et al. 2013). Failure of a neutral model to fit empirical data can also be revealing. To take one example, Gilbert et al. (2006) used a neutral model to predict the effects of forest fragmentation on an Amazonian tree community. It was found that the model accurately predicted the number of local extinctions, but failed to predict rates of change in species composition, highlighting that species life histories (incorporating factors such as matrix tolerance) are a vital consideration when determining the impacts of fragmentation on biodiversity (for further examples, see Rosindell et al. 2012).

Work on neutral theory has provided a new lens through which to view SADs and other primary macroecological patterns, and has provided the stimulus for increased debate on SADs in general (Chave 2004), particularly the mathematical methods used to test and investigate them. This has led to an increased recognition of stochastic models in ecology as an alternative to the deterministic models that have dominated the literature. Furthermore, much of the criticism directed at the neutrality assumption is misguided, as it in fact relates to the speciation mode (i.e., point mutation) incorporated in the early SINMs (Rosindell et al. 2010). Switching point mutation with the more realistic protracted speciation mode has been found to greatly improve predictions, such as species lifetimes, which did not previously match empirical data (e.g., Ricklefs 2003, 2006), while retaining the fit to SAD data.

A look to the future raises the possibility that the incorporation of neutrality within niche theory research may be less contentious, as while many view neutral- and niche-based theories as incompatible, this is not the case. The predominant view that niche and neutral processes are mutually exclusive is a “false dichotomy” (Leibold and McPeek 2006; Adler et al. 2007) and ignores the possibility that both types of processes act to structure communities concurrently; a fact acknowledged by Hubbell (2001, p. 24) himself. As Alonso et al. (2006, p. 455) contend, it is likely that the ecological reality is somewhere in between: “ecological communities are not often neutral, but they are not strictly hierarchical competitive communities either,” – a position closer to weak neutrality than hard neutrality, reflecting both niche-related processes and neutrality. To put it another way, community structure can be viewed as a continuum of combinations of stabilizing niche mechanisms and fitness differences between species. In this context, neutral models are just a special case in which fitness of all species is equal and there are no stabilizing niche effects (Adler et al. 2007). For instance, it has been shown that neutrality increases with species richness in ecological systems (Gravel et al. 2006; Bar-Massada et al. 2014), ostensibly due to increased niche overlap, as opposed to increased niche packing, with increasing richness. To take another example, Chust et al. (2013) used the method of Jabot and Chave (2011, see Table 3), incorporating the SAD to show that marine phytoplankton communities are structured through both niche and neutral assembly processes. In the midst of the niche versus neutrality debate, several studies have looked at ways of unifying the two perspectives (Bonsall et al. 2004; Tilman 2004; Hubbell 2006; Leibold and McPeek 2006; Gravel et al. 2006; Scheffer and van Nes 2006; Adler et al. 2007; Chisholm and Pacala 2010; Siepielski et al. 2010; Haegeman and Loreau 2011; Vergnon et al. 2012; Bar-Massada et al. 2014).

Work on reconciling neutral and niche theory has taken the form of both quantitative and nonquantitative models. In relation to the former, Chisholm and Pacala (2010) proposed an SINM which incorporates niches. Within the model, all the species in the metacommunity are assigned to a particular niche; but within each niche species behave neutrally. Using this model, they predicted SADs that approximate those predicted by a standard neutral model in high-diversity communities. This is intriguing as it suggests neutral processes underpin the SAD even when a community has strong niche structure. However, it has since been argued that the model of Chisholm and Pacala (2010) is flawed as it considers species to be largely independent (i.e., noninteracting) and is thus basically just a form of neutral model (Haegeman and Etienne 2011; Haegeman and Loreau 2011).

Tilman's (2004) stochastic niche theory is an earlier example of such an approach, offering an interpretation of community structure resolving some of the debate surrounding niche and neutral-based theories. Tilman proposed a theory having a basis in niche apportionment and resource use, but combining this with stochastic processes. It incorporates the ideas of dispersal and chance, and demographic stochasticity inherent within neutral theory, but equally emphasizes the importance of competition and resource division. Tilman (2004) concludes by highlighting that both his stochastic niche theory and neutral theory will produce similar SAD curve shapes and thus it is the underlying assumptions that are important: stochastic niche theory assumes a correlation between traits and abundances, and environmental context, whereas neutral theory assumes no such correlation. While a useful start point, Tilman's (2004) stochastic niche theory is not a “complete reconciliation” between competition and neutral theories as it does not incorporate the effects of variation in immigration and dispersal limitation (Gravel et al. 2006). To circumvent this issue, Gravel et al. (2006) proposed a model unifying Tilman's theory with the dispersal element of neutral theory, resulting in a continuum from niche to full neutrality.

It has also been shown empirically that the coexistence of ecologically equivalent species is not discordant with traditional niche-based theories. In an interesting test, Siepielski et al. (2010) manipulated the relative species abundance of two phylogenetically distant species of *Enallagma* damselflies and found evidence of ecological equivalence. However, previous work on *Enallagma* and the sister genus *Ischnura* had found strong evidence of ecological differentiation between species in the two genera, which enabled coexistence in the same food web. Thus, taken together these findings seemingly indicate that communities can be structured through both niche- and neutral-based processes (Siepielski et al. 2010; see also Leibold and McPeek 2006). However, given that we are unable to measure the full niche hyperspace volume, a conclusion of ecological equivalence has to be interpreted carefully.

**Figure 3:**
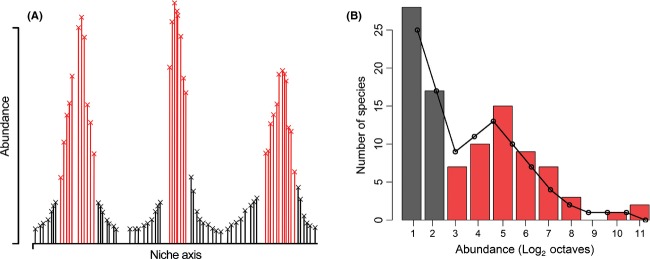
An illustration of how emergent neutrality can lead to multimodal species abundance distributions. (A) represents the abundance of a set of species as a function of a hypothetical niche axis. The species which comprise the peaks (red bars) within panel (A) are the abundant species in the community and correspond to the abundant species in the multimodal distribution (B; red bars). The species in the troughs (black bars) of panel (A) are relatively rare and correspond to the black bars in panel (B). The combination of these two sets of species in a sample results in a bimodal abundance distribution. The observed data used to construct (B) (colored bars) are from a sample of arthropod species in a fragment of native Laurisilva forest in the Azores (P.A.V. Borges, personal communication). A two-mode Poisson lognormal distribution has been fitted to the data (black line) using the functions in Dornelas and Connolly (2008). These data are used simply to provide an example of a multimodal species abundance distribution; the role of emergent neutrality in this particular system is unclear.

To take one final example, the recent advancement of emergent neutrality (EN) theory (originally termed “self-organized similarity”), that is, a situation in which neutrality is the outcome of community evolution, has placed a renewed focus on unifying niche and neutral perspectives in ecology. EN is based on Lotka-Volterra type competition models and describes a situation in which a community of competing species is clumped into groups of coexisting species along a niche axis (Scheffer and van Nes 2006; Segura et al. 2011; Vergnon et al. 2012; see also Holt 2006). According to the EN model, each species is located at some point along the niche axis (Fig. 3A), and species closer together experience a greater degree of interspecific competition. Through time the impact of this interspecific competition may lead to reduced survivorship and thus to a shift in the position of species along the niche axis away from the area of greatest competition. The counter-intuitive outcome is to create a packing effect in which species are grouped into sets of “self-organized modes that contain multiple coexisting species.” (Vergnon et al. 2012, p. 2). Within each self-organized mode, species are essentially functionally equivalent (neutral); it is demographic stochasticity and immigration into the community, rather than species interactions, which are the dominating processes acting within the modes (Barabás et al. 2013). This coexistence is exhibited for thousands of generations and the theory is applicable to multiple systems as long as the community is species-rich and there is a meaningful degree of niche overlap (Scheffer and van Nes 2006).Thus, EN theory can be seen as incorporating ecological asymmetric theories, combining both ecological and evolutionary processes, and both niche and neutral perspectives; the end point being clusters of self-similar species (Segura et al. 2011). Early tests of EN have produced promising results that should provide a catalyst for future research (Segura et al. 2011; Vergnon et al. 2012; but see Barabás et al. 2013 for a critique of the EN model).

In relation to SADs, EN theory provides an explanation of multimodality (Vergnon et al. 2012; but see Barabás et al. 2013). While the possibility has long been recognized, it has become increasingly apparent that many SADs may in fact be multimodal; that is, characterized by multiple distinct modes (Ugland and Gray 1982; Dornelas and Connolly 2008; Matthews et al. 2014). EN predicts a bimodal SAD as the species within the “core” of the niche axis modes are all relatively abundant (Fig. 3), whereas the species in the “valleys” of the niche axis modes, termed the “outsiders,” are relatively rare (Vergnon et al. 2012). EN theory can also account for multimodal SADs if abundances of species within the different niche axis modes significantly differ (Fig. 3A). While neutral simulations have predicted multimodal SADs (Borda-de-Água et al. 2007; Barabás et al. 2013), it has been argued that the patchy record of empirical tests of the ZSM (Table 3) means that EN provides a more “credible” interpretation of multimodal SADs than does the ZSM (Vergnon et al. 2013). That being said, a number of non-neutral mechanisms have been argued to underpin multimodal SADs (e.g., Magurran and Henderson 2003; Matthews et al. 2014), and it has been argued that multimodal SADs do not represent unequivocal evidence for emergent neutrality theory (Barabás et al. 2013).

## Future Research Avenues

Analysis of the SAD is a good test of neutral theory (Alonso et al. 2006). However, early use of the ZSM distribution predicted by SINMs was hampered by the lack of a full analytical solution. The publication of the sampling formula and maximum likelihood function (e.g., Etienne 2005) means it is now possible to fit the ZSM and compare it with other distributions (but see discussion below). The development of more spatially realistic SENMs (e.g., Rosindell and Cornell 2013) presents additional predicted SADs, which can be used in this endeavor. SENMs are an exciting advancement for neutral theory and SAD research more generally for two main reasons. First, they provide a form of neutral theory that predicts more realistic SADs. Second, the availability of SENMs removes the two-tier community structure underpinning the SINM (Fig. 1) and thus allows exploration of the SAD at multiple scales. The spatial scaling of SADs is an exciting area of current SAD research (e.g., Borda-de-Água et al. 2012) and it should be informative to gain insights from a neutral perspective in this ongoing discussion.

Prior to the development of SENMs, work on neutral theory and the SAD was stifled by the niche versus neutrality debate. In order to move forward, we need to set out a new research agenda synthesizing the two perspectives. Niche and neutral theories are not mutually exclusive; rather, they consider complementary processes. As such, increased insight into one theory aids in the comprehension of the other (Adler et al. 2007).

Within this new research agenda, it will be necessary to move away from the polarizing debate in which niche models are “pitted” against neutral models and the fit of predicted diversity patterns compared. Such an approach is based on the false dichotomization of the two theories as wholly conflicting explanations of community dynamics. This has been shown to be an inaccurate portrayal of the ecological reality in certain cases (e.g., Siepielski et al. 2010), illustrating the need to move to a layered approach in which in the roles of both niche and neutral processes are considered in tandem (e.g., Gravel et al. 2006; Vergnon et al. 2012). It also requires a shift away from the current approach in which evidence of neutrality is used as a beating stick with which to argue against any role of niche structure, and vice versa (Siepielski et al. 2010). Rather, we need to work toward better integrating neutral models with classical niche coexistence models to understand the processes underpinning SADs, in addition to other macroecological patterns. Such an approach will also have applied benefits. For example, niche and neutral theories have different implications for biodiversity conservation and a unified framework may aid in translating ecological theory to conservation practice (Holt 2006).

A focus on integrated models will require careful consideration of the speciation mode as this is integral to determining the ecological equivalence of species within a particular taxon (Leibold and McPeek 2006). Integrated models will also need to vary the assumptions of classical niche and neutral theories. It is likely that the ecological reality involves communities with an underlying niche structure, but with functional groups within the community that contain several ecologically similar species (Siepielski et al. 2010). For example, as Haegeman and Etienne (2011, p. 962) note, answering the question of how niche and neutral processes interact requires models which relax the condition of species independence (i.e., noninteraction) but in which species “interact differently when they belong to the same niche than when they belong to different niches.”

In pursuit of this aim of integration, it will also be necessary to consider approaches in addition to those involving the SAD. This is particularly pertinent for the SINM as analysis of the likelihood surface of the parameters *m* and *θ* has revealed a ridge where the likeliness is relatively homogenous (Etienne and Alonso 2007). We want to determine the relative importance of niche and neutral processes in structuring communities, that is, at which point along the aforementioned continuum of community structure particular communities lie (Gravel et al. 2006; Adler et al. 2007; Bar-Massada et al. 2014). As SADs are often unable to discriminate between different underlying processes (above), the comparison of SAD models may have limited power in certain cases (Leibold and McPeek 2006). Ultimately, we will require large-scale manipulative experiments to provide the answers to some of these questions (Adler et al. 2007). Nonetheless, SADs still have an important role to play. It is simply that to move forward we need to use abundance data to answer questions other than whether the ZSM fits the BCI data better than competing SAD models. For instance, the prediction of multimodal SADs (Matthews et al. 2014) is one of the strengths of EN theory (above).

Aside from unifying niche and neutral perspectives, neutral models and the SADs they predict can be used to gain new insights into, and make new predictions relating to, numerous classical ecological theories. For instance, a recently published neutral model of island biogeography (Rosindell and Phillimore 2011; see also Rosindell and Harmon 2013) builds on MacArthur and Wilson's (1967) ETIB. It produced many of the same predictions as the ETIB, such as a decrease in immigration with increasing island isolation, but also made several new predictions. For example, whereas abundance was not explicitly incorporated in MacArthur and Wilson's (1967) original model, the neutral model predicts the SAD of species on islands of varying isolation from the mainland. Finally, while it has not been extensively discussed in this review, one of the assets of neutral theory is that it predicts many ecological and biogeographical patterns (e.g., SARs; Rosindell and Cornell 2007), not just SADs. Thus, it is important that future research, particularly, the development of novel neutral models, places increased focus on the prediction of these other patterns. It is equally important that these developments incorporate recent advances that have increased the realism of neutral theory, such as protracted speciation.

## Conclusions

In the 13 years since Hubbell (2001) published his monograph, great strides have been made in developing a robust and analytical neutral theory for ecology. In relation to SADs, this has seen the derivation of an analytical solution for the ZSM with an associated sampling theory (Etienne 2005), the use of novel approaches to determine the goodness of fit of SAD models (e.g., Etienne and Olff 2004) and the development of SENMs (e.g., Rosindell and Cornell 2013). Wider application of neutral theory has also been witnessed, along with application of the ZSM beyond the theoretical domain to actual ecological problems (e.g., Latimer et al. 2005); although this has not been without controversy. Due to the underlying assumptions, neutral theory and especially the ZSM have received heavy criticism (e.g., Ricklefs 2003). However, falsifying these assumptions does not render the theory invalid in all contexts. Neutral theory in its entirety may be a simplification of ecological reality and its assumptions may often be violated in real natural systems, but its utility lies in its simplicity. Out of this controversy have arisen new perspectives on neutral theory and SADs, with a greater focus on stochastic processes. Several authors (e.g., Tilman 2004; Gravel et al. 2006; Vergnon et al. 2012) have worked to develop new theories integrating aspects of both neutral- and niche-based theories. These theories have then been used to make predictions and explain patterns in SADs. Advancement of models acting to unify niche and neutral perspectives in ecology represents a promising avenue for future research in neutral theory and the SAD, and ultimately will provide new insights in the search for a mechanistic explanation of SADs.
